# The Marble-Hand Illusion

**DOI:** 10.1371/journal.pone.0091688

**Published:** 2014-03-12

**Authors:** Irene Senna, Angelo Maravita, Nadia Bolognini, Cesare V. Parise

**Affiliations:** 1 Faculty of Psychology, Milano-Bicocca University, Milan, Italy; 2 IRCCS Istituto Auxologico Italiano, Milan, Italy; 3 Max Planck Institute for Biological Cybernetics and Bernstein Center for Computational Neuroscience, Tübingen, Germany; 4 Cognitive Neuroscience Department and Cognitive Interaction Technology-Center of Excellence, Bielefeld University, Bielefeld, Germany; ICREA-University of Barcelona, Spain

## Abstract

Our body is made of flesh and bones. We know it, and in our daily lives all the senses constantly provide converging information about this simple, factual truth. But is this always the case? Here we report a surprising bodily illusion demonstrating that humans rapidly update their assumptions about the material qualities of their body, based on their recent multisensory perceptual experience. To induce a misperception of the material properties of the hand, we repeatedly gently hit participants' hand with a small hammer, while progressively replacing the natural sound of the hammer against the skin with the sound of a hammer hitting a piece of marble. After five minutes, the hand started feeling stiffer, heavier, harder, less sensitive, unnatural, and showed enhanced Galvanic skin response (GSR) to threatening stimuli. Notably, such a change in skin conductivity positively correlated with changes in perceived hand stiffness. Conversely, when hammer hits and impact sounds were temporally uncorrelated, participants did not spontaneously report any changes in the perceived properties of the hand, nor did they show any modulation in GSR. In two further experiments, we ruled out that mere audio-tactile synchrony is the causal factor triggering the illusion, further demonstrating the key role of material information conveyed by impact sounds in modulating the perceived material properties of the hand. This novel bodily illusion, the ‘Marble-Hand Illusion', demonstrates that the perceived material of our body, surely the most stable attribute of our bodily self, can be quickly updated through multisensory integration.

## Introduction

An accurate knowledge of the material properties of our body is essential for adaptive and successful behavior, yet little is known on how the brain achieves such knowledge. Whenever grasping an object, for example, the velocity of reaching and the grip force should be carefully adjusted according to the material of the object and of our body. In order to perceive our body and the world around us, the brain constantly combines multiple sources of incoming sensory information with prior knowledge retrieved from memory [1]. However, unlike most bodily properties that frequently change over time (like posture and position), the material of our body never changes, so in principle it would be unnecessary for the brain to constantly try to infer it, as under normal circumstances all the senses would always provide the same information.

In the present study we set out to investigate whether the brain can update its knowledge about the material properties of the body by inducing an illusory perception of the material of the hand. We gently hit participants' right hand with a small hammer, and manipulated the auditory feedback so that each time the hammer hit the hand, participants heard the sound of a hammer against a stone. Impact sounds provide reliable cues about an object's material properties, and it is well-known that humans are readily able to infer the material of an object just by hearing it bounce [2]. An altered perception of the material of our body as a result of such a manipulated auditory feedback, would demonstrate that the brain does not take for granted the material of our body, and constantly updates its knowledge about our body's most stable property. Using a combination of subjective ratings and physiological measures, here we demonstrate that after a few minutes of such stimulation, participants experienced an altered perception of the material properties of their hands in the direction of the manipulated auditory feedback. This novel effect, the Marble-Hand illusion (MHI), demonstrates that our assumptions about the material of our bodies are built on the fly based on sensory information, and that even impact sounds of non-biological materials can quickly feel as originating from our own bodies.

## Results

### Experiment 1

**Figure 1:**
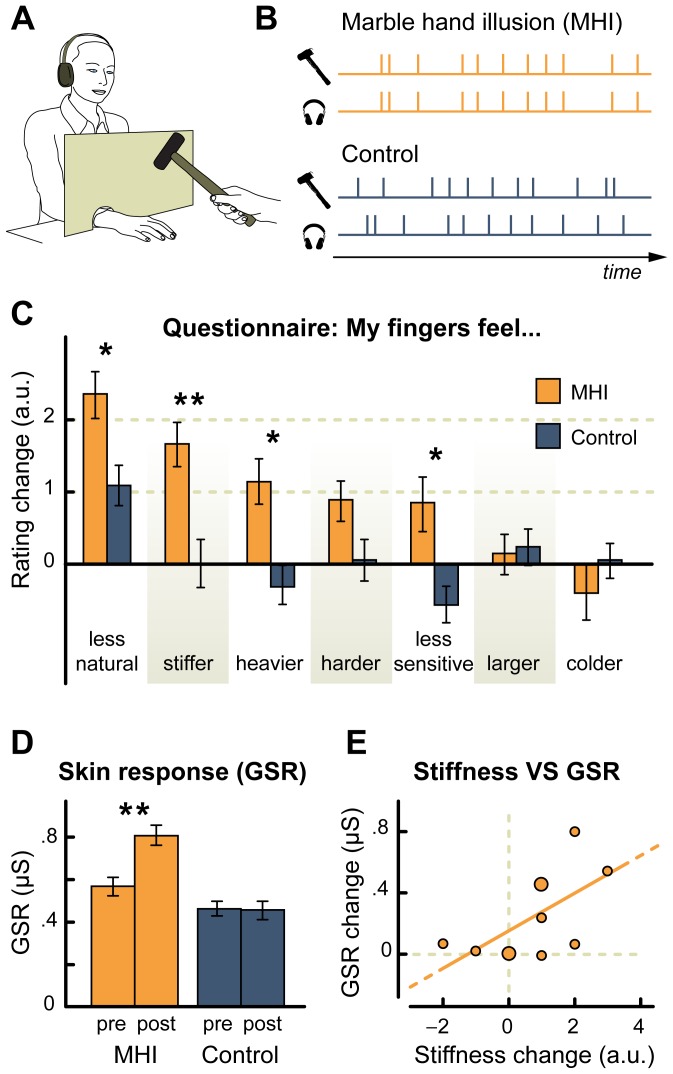
Experimental setup and results of Experiment 1. A. Schematic representation of the experimental apparatus. The opaque screen occluded the vision of the hand, but not of the approaching hammer. B. Temporal structure of hammer hits and impact sounds in the MHI and in the control condition. C. Questionnaire results. Scores represent the changes in the response between the post-stimulation and pre-stimulation presentations of the questionnaire. Error bars represent the standard error of the mean, and the asterisks indicate a significant difference (one asterisk p<0.05; two asterisks p<0.01) between the MHI and the control condition. The items of the questionnaire were presented in a random order, and participants provided their responses on a 7-points scale. D. Galvanic skin response results. After the stimulation, the response to threatening stimuli increased for the MHI (p<0.01), but not for the control. Error bars represent the standard error of the mean. E. Correlation between stiffness rating and Galvanic skin response in the MHI group (Pearson's ρ = 0.6; p = 0.02). The data is fitted with a Deming regression line; larger dots represent two points falling in close proximity.

Twenty-three naïve participants seated with their forearms resting on a table, and with their right hand hidden by an opaque screen (Figure 1a). To experimentally induce the MHI, an electrode was first connected to the participant's right hand, and another to a small hammer held by the experimenter. Each time the hammer hit the electrode on the hand–roughly once every 1.2 s in a non-rhythmic fashion–participants heard, via headphones, the sound of a hammer hitting a stone.

To quantitatively measure the perceptual correlates of the MHI, participants filled in a questionnaire before and after the stimulation. The questionnaire items assessed participants' hand perception in terms of stiffness, heaviness, hardness, temperature, naturalness, sensitivity, and size. Variations between pre- and post- stimulation responses were taken as evidence for the MHI. After the stimulation, several participants spontaneously described their right hand as feeling heavier, harder, and stiffer. They also reported feelings of numbness, pins and needles, and a lack of sensitivity, sometimes extending over the entire forearm (Table 1). Importantly, those spontaneous self-reports were confirmed by the results of the questionnaire, where the right hand was rated as being heavier, harder, stiffer, and less natural after the stimulation (Figure 1c; Table 2).

**Table 1:** Sensations reported spontaneously after the stimulation.

Spontaneously reported sensations	# occurrences	
	MHI	Controls
My hand feels numb (e.g., insensitive, with pins and needles)	10	0
My hand feels stiffer	9	2
My hand feels heavier	6	0
My hand feels colder	6	1
The feeling of stiffness and numbness seems to extend to the whole foreharm	5	0
My hand feels “different”	3	0
My hand feels lighter	0	3
My hand feels softer	0	4

**Table 2:** Mean difference and SEM in the questionnaire items before and after stimulation.

Questionnaire item *'my fingers feel'*	MHI (post-pre)	Controls (post-pre)	Z	p	r
Natural as usual - Unnatural	2.4±0.3 (p = 0.001**)	1.1±0.3 (p = 0.017*)	2.78	0.025*	0.58
Extremely flexible - Extremely stiff	1.7±0.3(p = 0.005**)	0±0.3 (p = 0.8)	3.26	0.007**	0.68
Extremely light - Extremely heavy	1±0.3 (p = 0.007**)	−0.3±0.3 (p = 1)	3.03	0.012**	0.63
Extremely soft - Extremely hard	0.9±0.3 (p = 0.039*)	0±0.3 (p = 1)	1.7	0.3	0.35
Extremely sensitive - Extremely insensitive	0.8±0.4 (p = 0.3)	−0.6±0.3 (p = 0.2)	2.71	0.028*	0.57
Extremely small - Extremely large	0.1±0.3 (p = 0.6)	0.2±0.3 (p = 1)	0.31	1	0.06
Extremely cold - Extremely hot	−0.4±0.4 (p = 0.6)	0±0.2 (p = 1)	−0.71	1	−0.15

The values in brackets relate to the difference between before and after the stimulation as assessed by the Wilcoxon test. Comparison between MHI and control was done using the Mann-Whitney's U test. The last column reports the effect size r. Asterisks indicate a significant difference between the MHI and the control condition (one asterisk p<0.05, two asterisks p<0.01). All p-values are corrected with Bonferroni-Holm correction.

A second group of 23 naïve participants was tested in a control condition, where hammer hits and impact sounds were temporally uncorrelated [3] (Figure 1b). This group did not spontaneously report any evidence compatible with the occurrence of the MHI (Tables 1–2). Furthermore, compared to the controls, participants in the MHI experienced their hand as less natural, stiffer, heavier and less sensitive (Figure1c, Tables 1–2). Although the difference in perceived hand hardness between the MHI and the controls just failed to reach significance, it is important to note that hardness rating significantly increased after the stimulation in the MHI, but not in the controls.

To substantiate the robustness of the effect over and above subjective reports, we measured how the Galvanic skin response to threatening stimuli varied during the MHI (see Methods session). Galvanic skin response provides a physiological measure of arousal induced by stimuli threatening the body, and it can be modulated by other bodily illusions [4], [5], [6], [7]. Therefore, we measured Galvanic skin responses to a needle approaching the right hand in a subset of participants in the MHI (N = 11) and in the control group (N = 11) before and after the stimulation procedure. Results showed that the Galvanic skin response to threatening stimuli significantly increased in the MHI (pre-stimulation mean = 0.56 μs, SD = 0.47; post-stimulation mean = 0.81 μs, SD = 0.5, p<0.01), but not in the control group (pre-stimulation mean = 0.46 μs, SD = 0.38; post-stimulation mean = 0.46 μs, SD = 0.45, Figure 1d). Notably, such increase in GSR in the MHI condition positively correlated with reported hand stiffness (r = 0.6, p = 0.02; Figure 1 e). That is, in line with previous bodily illusions also the MHI is associated with altered physiological arousal. Regarding the reason for the increase in GSR, we can only speculate. On one hand, if the hand feels harder it may also feel less vulnerable, hence the GSR should decrease, rather than increase. However, previous studies reported amplified pain sensations when uncertainty is added to an incoming noxious stimulus [8]. Therefore, it is possible that the misperceptions of the hand in the MHI may reduce the capability of participants to predict the potential danger of the upcoming threat, thus increasing autonomic alert responses to the needle. Also, the positive correlation between skin conductance and reported hand stiffness may reflect the fact that a stiffer hand would be less effective in escaping an impending threat, hence increasing physiological arousal.

Overall, such a GSR modulation would attest to a genuine alteration of body representation induced by the synchronous audio-tactile stimulation, more than any generic report bias due to the introspective nature of the questionnaire. Indeed, the modulation of skin conductance witnesses a modulation of the physiological response to threatening stimuli occurring at the level of the autonomic nervous system. Remarkably, such an increase in skin conductance positively correlated with the feeling of stiffness, hence demonstrating a relation between the phenomenology of the illusion and physiological response.

### Experiment 2

In the previous experiment, we measured the effects of the MHI by comparing a synchronous audio-tactile condition with an asynchronous audio-tactile condition. As a consequence, it may be argued that the results may be due to the mere synchrony of the auditory feedback in the MHI condition rather than to the material information conveyed by the impact sounds. Therefore we run two further control experiments using different auditory feedback, while maintaining audio-tactile synchrony.

**Figure 2:**
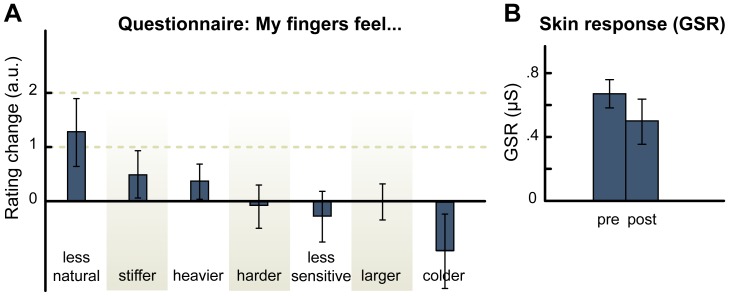
Results of Experiment 2. A. Questionnaire results. Scores represent the changes in the response between the post-stimulation and pre-stimulation presentations of the questionnaire. Error bars represent the standard error of the mean. The items of the questionnaire were presented in a random order, and participants provided their responses on a 7-points scale. B. Galvanic skin response (GSR) results. GSR was not affected by the stimulation (no significant difference in the GSR before and after the stimulation).

In Experiment 2, we adopted the same procedure of the MHI group of Experiment 1, with the exception that now the auditory stimulus was a pure tone (440 Hz, 25 ms) played in synchrony with the tactile stimulation. Pure tones do not provide any reliable cue to an object's material, therefore they should not affect subjective reports about hand perception nor physiological responses. Eleven participants took part in this experiment. Overall, they did not spontaneously report any altered feelings about the hand following the stimulation, nor they showed any change in their ratings at the questionnaire (all ps>0.5, Figure 2 a). Moreover, the Galvanic skin response to threatening stimuli did not significantly change (t = 1.54; p = 0.15) as a consequence of the audio-tactile stimulation (pre-stimulation mean = 0.67 μs, SD = 0.3; post-stimulation mean = 0.5 μs, SD = 0.45, Figure 2 b). The fact that a synchronous audio-tactile stimulation did not significantly affect subjective reports and threat-evoked GSR, when using a pure tone, rules out any interpretations of the results of Experiment 1 in terms of a generic effect of synchronous audio-tactile stimulation. This finding supports the conclusion that the results of Experiment 1 reflect a genuine bodily illusion induced by the material information conveyed by the hitting sounds.

### Experiment 3

To further exclude that the changes in ratings and GSR reported so far are due to the hammer hitting the hand irrespective of any manipulation of the auditory feedback, we run a third experiment in which participants simply heard the natural sound produced by the actual contact of the hammer against their hand.

**Figure 3:**
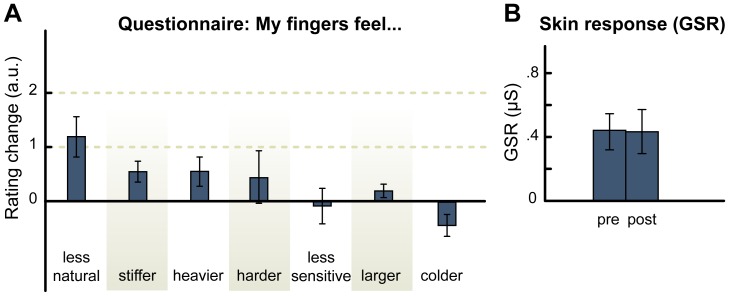
Results of Experiment 3. A. Questionnaire results. Scores represent the changes in the response between the post-stimulation and pre-stimulation presentations of the questionnaire. Error bars represent the standard error of the mean. The items of the questionnaire were presented in a random order, and participants provided their responses on a 7-points scale. B. Galvanic skin response (GSR) results. GSR was not affected by the stimulation (no significant difference in the GSR before and after the stimulation).

Eleven participants were tested in Experiment 3. The GSR was not affected by the audio-tactile stimulation (pre-stimulation mean = 0.45 μs, SD = 0.3; post-stimulation mean = 0.44 μs, SD = 0.45; t = 0.12, p = 0.9, Figure 3 b), and participants did not report any alteration in their hand perception, neither in the self-reports nor in the questionnaire (all ps>0.1, Figure 3 a). Taken together, the lack of any significant results in Experiment 2 and 3 suggest that to induce illusory perception of the material properties of the body, the feedback sound has to provide clear, unambiguous, cues to a specific material (such as marble).

## Discussion

When exposed to multisensory signals that correlate in time and space, but provide incongruent cues to body material, the brain can either keep those signals segregated, or else integrate them and resolve the incongruence by updating the perception of body material. The MHI demonstrates that the brain integrates correlated signals [9], and quickly updates the body schema, which consistently results in a vivid bodily illusion.

Most features of our body continuously change over time. For instance, the size of our body varies due to growth and posture, the seen and felt position of the hands can be offset by muscle fatigue and distorting lenses, and the texture of the skin varies with moisture. Because of this inherent variability, the estimate of such variables should be updated continuously, based on internal models and incoming sensory information [1]. Therefore, it is to some extent likely that simple experimental manipulations can reliably alter the perception of all such bodily attributes [3], [10], [11]. For instance, it has been demonstrated that an alien [3] or an invisible body part (obtained by either stroking a volume of empty space or a table-top) [7], [4] can be included in the body representation. However, the material of our body never changes; hence it would be superfluous for our brain to constantly monitor it. The MHI, instead, demonstrates for the first time that even the perception of such a time-invariant attribute is rapidly updated based on the incoming multisensory information.

Previous studies have already shown that sounds can alter body perception [12]. In the parchment skin illusion, for example, the perceived moisture of the skin can be altered by manipulating the sound of rubbing hands [11]. Nevertheless, such changes were still within the limits of biological plausibility, given that the human skin moisture can indeed be modulated by contingent factors. The MHI brings these consequences to the extreme, and demonstrates that even impact sounds of non-biological materials –like marble and metal– can be consistently attributed to the body, as if its core material could indeed be modified. This surprising perceptual plasticity might help to explain why tools and prostheses can so easily merge into our body schema, in spite of their material [5], [13], [14].

## Methods

In Experiment 1, to trigger the auditory stimuli, we attached a thin strip of metal foil tape to the right hand of the observers. The tape and the hammer were connected through a wire to a computer, and operated as a switch, so that each time the hammer touched the tape, a custom software based on the Psychtoolbox 3 [15] played the sound of a hammer hitting a marble surface. Due to processing delays, the average offset between the hit and the impact sound in the synchronous condition was 36 ms. Given the relatively low sensitivity to audio-tactile temporal delays [16], and adaptation to audio-tactile asynchrony [17], such a delay was not noticeable. The impact sound was embedded in a constant white noise. To maximize the illusion (e.g., see [18], [19]), the impact sound was ramped on gradually during the stimulation, being silent at the first hit, and fully audible within the first 30 hits. Participants were not informed of this manipulation, and were simply told that the noise continuously played by the headphones served to cancel out any external auditory disturbances. In the synchronous conditions, the sound was played immediately after the hit, whereas in the asynchronous condition, the sound was played with a delay randomized between 800 and 1600 ms.

To create spatialized sounds (that is, to simulate the source of the auditory stimuli in the 3D external space), we recorded the sound of a hammer hitting a marble stone using in-ear recording technology (see [9]). A pair of miniature microphones was placed inside the ear canal of a volunteer –which did not participate in the experiment– and we recorded the sound of a hammer hitting a piece of marble positioned next to the volunteer right hand. In order to enhance the realism, we recorded ten hitting sounds, so that during the experiment, at each hit the computer randomly selected and played one of these sounds. When played via headphones in the experimental setup, such sounds were perceived as coming from the same position as the right hand.

Before and after the stimulation, which lasted approximately 5 minutes, participants filled in a questionnaire written in Italian, the native language of all the observers, consisting of 7 items (see Table 2) aimed at measuring the subjective perception of the material of the hand. On a 7-point Likert scale, ranging from –3 to +3, participants had to rate how they felt their fingers along a series of dimensions marked by pairs of contrasting attributes (Table 2). Participants had to tick the number that better described their feeling on the scale. The order of the items was randomized across participants, and across sessions within participants. In Experiment 2, the procedure was the same as the MHI group in Experiment 1 (i.e. auditory stimulation synchronous with the tactile stimulation), but the sound was a pure tone (440 Hz, 25 ms of duration). In Experiment 3, there was no manipulation of the auditory feedback, and participants simply heard the natural sound produced by the impact of the hammer against their hand. In Experiment 3 we aimed at investigating whether subjective response and GSR modulation occur when hearing the natural sound produced by the contact of a hammer against the hand. Since in a pilot experiment we found that the recorded sound of a hammer against human skin was not reliably recognized when played through headphones and embedded in white noise, the setting was changed to a more ecological stimulation. That is, the auditory stimuli consisted of the physical impact sounds between the hammer and participant's skin with no further manipulations. Given the low intensity of such natural sounds, in a pilot study we ascertained that participants were able to detect and recognize them. Ten participants were blindfolded and performed 20 trials in which the experimenter gently hit her own hand with the hammer once or twice per trial, and participants had to report how many impact sounds they heard. Accuracy was around 84%, thus demonstrating that participants could indeed detect the sounds. Furthermore, at the end of the experiment 9 out of 10 participants correctly reported that the sound was produced by an object against human skin. Results of this pilot study demonstrate that, in spite of being quiet, the natural sound of a hammer hitting a hand was clearly detectable and recognizable. Moreover, all participants in Experiment 3 reported that they clearly heard the sound of the hammer against their hand throughout the experiment.

Overall, 68 right-handed healthy individuals participated in the three main experiments. In Experiment 1 we tested twenty-three participants per condition, for a total of forty-six participants (MHI group: 20 females, mean age = 25, SD = 4.1, range 19–31; Control group: 15 females, mean age = 25.4, SD = 3.7, range 19–35). Eleven participants also took part in Experiment 2 (6 females, mean age = 27.5, SD = 2.9, range 24–33), and Experiment 3 (9 females, mean age = 27.9, SD = 4, range 24–35).

### Assessment of Galvanic skin response

Before and after the experimental stimulation (namely, after filling in the first questionnaire, and before filling in the second questionnaire), galvanic skin response (GSR) to threatening stimuli was recorded in a subset of participants in the MHI group (N = 11) and in the control group (N = 11) in Experiment 1. GSR was recorded in the same number of participants in Experiment 2 (N = 11) and 3 (N = 11). Two Ag–AgCl electrodes (1081FG Skin Conductance Electrode) with constant voltage (0.5 Volt) were attached to subjects' proximal phalanges of the index and middle fingers of their left hand (the non-stimulated hand). At the beginning of each trial, the experimenter held the needle under the table, then lift it above the table-top and moved it toward participant's right index finger, and eventually threatened the participant twice, by approaching the needle to the same finger, but without touching it (see [20]). Given that the participant's hand was concealed behind an opaque screen, the participants could only see the approaching needle, but not its contact with the hand. We then analyzed GSR responses to such simulated stimulations. The reason for using simulated, instead of real, noxious stimuli, was to minimize somatosensory cues from the hand, which could have quickly cancelled any feeling induced by the audio-tactile training with the hammer. This procedure was performed before and after the hammer stimulation. In order to prevent habituation to needles threatening the hand, without touching it, immediately after measuring the GSR, and before inducing the illusion, we touched the finger of the participants with the needle one more time. Participants were asked to relax, and carefully watch the approaching needle. Each threatening stimulus was presented after the GSR signal returned to a resting-state level, based on visual inspection. To further minimize interference across GSR measurements, the minimal interval between the presentations of two consecutive threatening stimuli was 10 s at least.

GSR was recorded with a SC 2071 device (Bioderm, UFI, Moro Bay, California) following standard guidelines [21]. Saline conductor gel was used to improve the signal to noise ratio. The GSR recording was digitalized and sent to a PC for further analyses. The gain parameter was set at 10 μS/Volt and the A/D resolution was 12bit, which gave a recordable response ranging from 0.1 to 100 μS. The sample rate was set at 10 Hz.

For each threatening stimulus, we calculated the GSR [22], as the difference between the maximum skin conductance (measured in a time window of 6 seconds after the appearance of the threatening stimulus approaching the hand) and the baseline, (measured as the mean value before the stimulus within a window of 0.3 seconds). This procedure was repeated twice, and the two measurements were averaged.

### Data Analysis

To measure how the stimulation changed the subjective reports about hand perception, as assessed with the questionnaire, for each item we calculated the difference between the second and the first presentation of the questionnaire (i.e., after and before the audio-tactile stimulation, respectively). The statistical significance of the difference in the responses between the second and first presentation of the questionnaire was calculated using the Wilcoxon Signed rank test in Experiment 1, 2 and 3. Moreover, in Experiment 1 a Mann-Whitney U test was used to test for difference between the MHI and the control groups and we then calculated the effect size using the 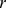 statistics (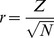, where 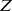 is the output of the Mann-Whitney's test, and N is the total number of observations, e.g., see [23]). Note that conventionally effects size larger than 5 are considered large, and in the present case all of the significant effects had effect sizes larger than 5. Results are reported in Table 2. For all tests, we applied a Bonferroni-Holm correction for multiple comparisons across all items.

To test for effects of the MHI on skin conductivity, in Experiment 1 the GSR data was submitted to an analysis of variance (ANOVA) with order of presentation (before and after the stimulation) as within-participant factor, and type of stimulation (synchronous vs asynchronous) as between-participants factor. None of the main effects reached statistical significance (type of stimulation: F_1,20_ = 1.47, p = 0.24; order of presentation: F_1,20_ = 4, p = 0.06), however there was a significant order by type of stimulation interaction (F_1,20_ = 4.58; p<0.04, total η^2^ = 0.23), indicating that the GSR changed as a function of both order of presentation and type of stimulation. Post-hoc comparisons using the Newman-Keuls test revealed a significant difference between the first and the second GSR measure in the MHI group (p<0.01) but not in the control group (all other comparisons did not reach statistical significance, ps>0.21).

Pearson's correlation analyses were performed to measure the association between the GSR and the magnitude of the illusion, i.e., the change in GSR before and after the stimulation and the changes in the responses to the questionnaires before and after the stimulation. GSR positively correlated with the subjective report of stiffness (r = 0.6; p<0.02), indicating that the more participants felt their hand to be stiff, the more their GSR to threatening stimuli increased. Notably, taking into account only the subset of participants who spontaneously reported altered sensations from the stimulated hand (see Table 1), such correlation increased to r = 0.88. Other items did not correlate with the GSR.

In Experiment 2 and 3, paired t-tests were used to compare the GSR data before and after the stimulation. None of the paired t-tests reached significance (Experiment 2: t = 1.54; p = 0.15; Experiment 3: t = 0.12, p = 0.9), demonstrating that in Experiment 2 and 3 GSR was not affected by the audio-tactile stimulation.

Additionally, we run further analyses to directly compare results between the MHI condition of Experiment 1 and Experiments 2 and 3. Such analyses are reported in the File S1.

### Ethics Statement

This study was conducted in accordance to the declaration of Helsinki, and had ethical approval from the Department of Experimental Psychology at the University of Milano-Bicocca. All participants provided written informed consent and received course credits in return.

## Supporting Information

File S1 **Supplemental analyses.**
(DOCX)
