# Supplementary material for: The Marble-Hand Illusion
Source: PLoS One. 2014 Mar 12;9(3):e91688. doi: 10.1371/journal.pone.0091688 (PMC3951417; doi:10.1371/journal.pone.0091688)
Supplement: File S1 — Supplemental analyses. (DOCX) [file pone.0091688.s001.docx]

**Supporting information**

S1. Supplemental analyses

Given that Experiments 2 and 3 have only one condition and they are control experiments for the synchronous (i.e. MHI) group of Experiment 1, for completeness we decided to run supplemental analyses to directly compare the post-pre differences in Experiment 2 and 3 with the differences in the MHI group of Experiment 1.

**Questionnaire.** Compared to Experiment 2, in the MHI group of Experiment 1 participants felt the hand significantly stiffer (Mann-Whitney U test: U=3.07; Z=3.14, p=0.0017) and harder (U=74.5; Z=1.97, p=0.049); the other items did not differ significantly. Also, participants in the MHI group in Experiment 1 felt the hand stiffer (U=47; Z=3.03, p=0.0024) compared to participants in Experiment 3. Additionally, in Experiment 1, during the MHI participants felt the hand slightly more unnatural than in Experiment 3, though this difference was just nearly significant (U=74.5; Z=1.95; p=0.0507). Other items did not differ significantly.

**GSR.** In a similar fashion, we compared GSR data between the MHI group in Experiment 1 and Experiment 2 by using an analysis of variance (ANOVA) with Order of presentation (before and after the stimulation) as within-participant factor, and Type of stimulation (MHI Experiment 1vs Experiment 2) as between-participants factor. Results showed a significant Order by Type of stimulation interaction (F1,20=8.7; p=0.0078, total η^2^=0.44), indicating that GSR changed as a function of both order of presentation and type of stimulation. Post-hoc comparisons using the Newman-Keuls test revealed a significant difference between the first and the second GSR measure in the MHI group only (p=0.039), but not in the Experiment 2.

The same analysis was conducted to compare MHI group with Experiment 3. Results showed a significant Order by Type of stimulation interaction (F1,20=6.02; p=0.02, total η^2^=0.3) and post-hoc tests showed that the difference between before and after the stimulation was significant only for the MHI group (p=0.003).

Despite the methodological differences between the three experiments, which imply a number of confounds that may hamper the interpretation of the results of their direct comparison (e.g., in Experiment 2, being pure tones, stimuli to were not spatialized; in Experiment 3 on top of using different sounds we did not use headphones nor we played background white noise, and impact sounds were very quiet), all in all, these additional analyses reveal a significant difference in stiffness rating and GSR between the synchronous condition of Experiment 1 and the control experiments. Given that stiffness rating is the primary phenomenological correlate of the present illusion, and the only item that correlates with skin response, these results by and large support the conclusions outlined in the main text.
